# A novel cripavirus of an ectoparasitoid wasp increases pupal duration and fecundity of the wasp’s *Drosophila melanogaster* host

**DOI:** 10.1038/s41396-021-01005-w

**Published:** 2021-05-18

**Authors:** Jiao Zhang, Fei Wang, Bo Yuan, Lei Yang, Yi Yang, Qi Fang, Jens H. Kuhn, Qisheng Song, Gongyin Ye

**Affiliations:** 1 State Key Laboratory of Rice Biology & Ministry of Agriculture Key Lab of Molecular Biology of Crop Pathogens and Insects, Institute of Insect Sciences, Zhejiang University, Hangzhou, Zhejiang Province China; 2 Integrated Research Facility at Fort Detrick, National Institute of Allergy and Infectious Diseases, National Institutes of Health, Fort Detrick, Frederick, MD USA; 3 Division of Plant Sciences, College of Agriculture, Food and Natural Resources, University of Missouri, Columbia, MO USA

**Keywords:** Virus-host interactions, Animal physiology

## Abstract

We identified a 9332-nucleotide-long novel picornaviral genome sequence in the transcriptome of an agriculturally important parasitoid wasp (*Pachycrepoideus vindemmiae* (Rondani, 1875)). The genome of the novel virus, Rondani’s wasp virus 1 (RoWV-1), contains two long open reading frames encoding a nonstructural and a structural protein, respectively, and is 3’-polyadenylated. Phylogenetic analyses firmly place RoWV-1 into the dicistrovirid genus *Cripavirus*. We detected RoWV-1 in various tissues and life stages of the parasitoid wasp, with the highest virus load measured in the larval digestive tract. We demonstrate that RoWV-1 is transmitted horizontally from infected to uninfected wasps but not vertically to wasp offspring. Comparison of several important biological parameters between the infected and uninfected wasps indicates that RoWV-1 does not have obvious detrimental effects on wasps. We further demonstrate that RoWV-1 also infects *Drosophila melanogaster* (Meigen, 1830), the hosts of the pupal ectoparasitoid wasps, and thereby increases its pupal developmental duration and fecundity, but decreases the eclosion rate. Together, these results suggest that RoWV-1 may have a potential benefit to the wasp by increasing not only the number of potential wasp hosts but also the developmental time of the hosts to ensure proper development of wasp offspring.

## Introduction

Arthropods are among the most abundant invertebrates on the planet. An improved understanding of arthropod viruses could lead to novel measures for protection of agriculturally beneficial insects, such as western honey bees (*Apis mellifera* Linnaeus, 1758), from viral infection [1]. Equally important, arthropod viruses could be used as biocontrol agents to infect and suppress pestiferous insects, such as codling moths (*Cydia pomonella* (Linnaeus, 1758)) and velvet bean caterpillars (*Anticarsia gemmatalis* Hübner, 1818) [2], or arthropod vectors of plant, animal, and human pathogens [3–5]. Recent advances in viral metagenomics and high throughput sequencing technologies have greatly facilitated invertebrate RNA virus discovery [6–9], thereby greatly increasing the possibilities for arthropod–virus interactions to control arthropod pests, or vice versa, thus strengthening beneficial arthropods.

Parasitoid wasps (order Hymenoptera) are the most common type of parasitoid insects. These wasps lay their eggs inside or on the bodies of other arthropods, which ultimately serve as food for the developing wasp larvae after hatching [10]. Therefore, parasitoid wasps are enemies of other arthropods and play an important role in biological control of pests [11–13]. The DNA virome of parasitoid wasps has been characterized for many years [14, 15]. However, the RNA virome of parasitoid wasps is still poorly understood.

The first virus resembling members of the positive-sense RNA virus order *Picornavirales* in braconid *Microplitis croceipes* (Cresson, 1872) parasitoid wasps was reported in 1992 [16]. Subsequently, picornaviral RNA-directed RNA polymerase (RdRp) nucleic acids in ichneumonidae *Venturia canescens* (Gravenhorst, 1829) [17] and pteromalid *Nasonia vitripennis* (Walker, 1836) [18] wasps were reported in 2005 and 2010, respectively. In pteromalid *Pteromalus puparum* (Linnaeus, 1758) wasps, some detected nucleic acids were found to be homologous to the open reading frame (ORF) encoding the capsid protein of cricket paralysis virus (CrPV: *Picornavirales*: *Dicistroviridae*: *Cripavirus*) [19]. It is known that *N. vitripennis* wasps harbor Nora-like viruses (*Picornavirales*: unclassified) and iflavirids (*Picornavirales*: *Iflaviridae*) [18]. Yet, the effect, if any, of these viruses on their hosts has been unclear.

Pteromalid *Pachycrepoideus vindemmiae* (Rondani, 1875) wasps are cosmopolitan solitary ectoparasitoids that attack pupae of a range of muscomorph dipterans, such as common fruit flies (*Drosophila melanogaster* Meigen, 1830), medflies (*Ceratitis capitata* (Wiedemann, 1824)), cabbage flies (*Delia radicum* (Linnaeus, 1758)), lesser houseflies (*Fannia canicularis* (Linnaeus, 1761)), houseflies (*Musca domestica* Linnaeus, 1758), and tephritid fruit flies (*Rhagoletis* sp.) [20]. Consequently, *P. vindemmiae* wasps have been evaluated mainly for the control of stable and house fly populations [21–23]. In Europe and the United States, *P. vindemmiae* is one of only two parasitoid wasps that naturally and successfully attack and kill the spotted wing *drosophila*, *Drosophila suzukii* (Matsumura, 1931) [24, 25], which is a major agricultural concern, as it infests ripening fruit rather than rotting fruit. *P. vindemmiae* wasps also attack melon flies (*Bactrocera cucurbitae* (Coquillett, 1849)) [26], a major pest of melons and related crops. Because of the ability to attack these two pests, *P. vindemmiae* wasps have great potential as a biological control agent.

Our research indicated that the venom of *P. vindemmiae* wasps significantly inhibits lamellocyte adherence and induces plasmatocyte death in *D. melanogaster* [27], and we characterized *P. vindemmiae* wasp venom composition [28, 29]. As part of this research, we sequenced the wasp’s transcriptome and discovered a novel cripavirus. Investigation of viral tissue distribution, developmental expression profile, transmission strategy, and direct impact on *P. vindemmiae* wasps revealed that the novel virus had no obvious effects on wasp but infects *D. melanogaster* and increases their pupal duration and fecundity, thus possibly providing the wasps with an increased number of pupae and extended pupal periods for parasitization.

## Methods

### Insect rearing

Insect rearing was performed based on previously reported methods [27]. Briefly, *D. melanogaster* were obtained from multiyear laboratory breeding colony *w*^*1118*^ and raised on standard cornmeal medium (1 L water, 105 g corn flour (Chongruifeng, Tieling, Liaoning Province, China), 75 g brown sugar (Ganzhiyuan, Nanjing, Jiangsu Province, China), 7.5 g agar (Macklin, Shanghai, China), 6.25 mL propionic acid (Aladdin, Shanghai, China), and 20 g yeast extract (Aladdin)) at 25 °C with 60 ± 5% relative humidity and a photoperiod of 16 h of light to 8 h of darkness (16:8 h light:dark). A colony of *P. vindemmiae* wasps was provided by Yongyue Lu (South China Agricultural University, Guangzhou, Guangdong Province, China) in January 2016 and subsequently maintained with *D. melanogaster* pupae at 25 °C with a photoperiod of 14:10 h (light:dark) as previously described [30]. After wasp emergence, adults were sealed in sterilized glass finger-shaped tubes (Hongtai Experimental Equipment, Nantong, Jiangsu Province, China) (18 × 82 mm) with sponges (Hongtai Experimental Equipment) and fed a 10% honey solution (v/v, honey/deionized distilled water). Wasps that had successfully parasitized *D. melanogaster* pupae were individually tested for the presence of Rondani’s wasp virus 1 (RoWV-1) using methods described previously [31]. Microscopy was used to detect old parasitoid larvae or pupae in *D. melanogaste*r pupae, which vary greatly in shape and are easy to recognize. The offspring of particular wasps in the RoWV-1 (–) or RoWV-1 (+) breeding pairs were maintained in RoWV-1 (–) or RoWV-1 (+) *D. melanogaster* pupae to produce the RoWV-1 (–) or RoWV-1 (+) colonies for this study. *D. melanogaster* that had successfully emerged were tested for the presence of RoWV-1 using the same methods used for *P. vindemmiae* wasps. The offspring of a particular *D. melanogaster* in the RoWV-1 (–) or RoWV-1 (+) breeding pairs was reared to produce the colonies for this study. The *D. melanogaster* RoWV-1 (+) colony maintained its virulent stability through contaminated food. RoWV-1 (–) and RoWV-1 (+) *D. melanogaster* and *P. vindemmiae* wasps were bred in two separate places to avoid accidental contamination.

### Virus genome sequencing

Primers for viral genome sequencing were designed based on the dicistrovirid-like contig (representing a genome of a virus here designated RoWV-1) discovered in the *P. vindemmiae* wasp transcriptional profiling (Supplementary Table S1). Total RNA was extracted from adult female *P. vindemmiae* wasps using TRIzol (Invitrogen, CA, USA). RNA concentrations of individual samples were measured using a Nanodrop 2000 (Thermo Scientific, Wilmington, DE, USA). Single-strand cDNA was synthesized from the RNA using the TransScript One-Step gDNA Removal and cDNA Synthesis SuperMix Kit (TransGen Biotech, Beijing, China). cDNA was used as a template for polymerase chain reaction (PCR). Virus genome termini sequences were confirmed by 5’ and 3’ rapid amplification of cDNA ends (RACE) using the SMART RACE cDNA amplification kit (Clontech, California, USA) according to the manufacturer’s instructions. Amplified PCR products were cloned into the pGEM-T Easy vector (Promega (Beijing) Biotech Co., Beijing, China) and sequenced as described previously [31].

### Phylogenetic analyses

Nucleotide sequence analysis and assembly were performed using DNAStar software version 5.02 (Madison, WI, USA). ORFs of the RoWV-1 genome were predicted using National Center for Biotechnology Information (NCBI) ORFfinder (https://www.ncbi.nlm.nih.gov/orffinder/). For each ORF, InterPro (http://www.ebi.ac.uk/interpro/) was used for prediction and analysis of protein structure and function [32]. Multiple alignments of amino acid sequences were performed using Clustal Omega (http://www.ebi.ac.uk/Tools/msa/clustalo/) and edited by Gene Doc (http://www.softpedia.com/get/Science-CAD/GeneDoc.shtml). The NCBI PAirwise Sequence Comparison (PASC) classification tool (https://www.ncbi.nlm.nih.gov/sutils/pasc/viridty.cgi?textpage=overview) was used for pairwise comparisons of viral genomes [33]. The phylogenetic tree was constructed using the maximum likelihood method and Poisson model with 1000-fold bootstrap resampling using MEGA X software (https://www.megasoftware.net/) [34]. Accession numbers of analyzed positive-sense RNA virus genomes are listed in Supplementary Table S2.

### Virus detection and quantification

To detect RoWV-1 in *P. vindemmiae* wasps, two pairs of primers (PVDA-1/PVDS-1 and PVDA-2/PVDS-2), amplifying 597-bp and 550-bp fragments, were designed based on the RoWV-1 genomic sequence (Supplementary Table S1). Total RNA from each sample was used to synthesize cDNA as described above. PCR was run as follows: 30 s at 94 °C, 30 s at 55 °C, and 45 s at 72 °C for 35 cycles. Amplifications were visualized by 1% agarose gel electrophoresis and ethidium bromide staining. The 40S ribosome of *P. vindemmiae* wasp was used to determine the quality of the cDNA by RT-PCR (Supplementary Table S1, Pv-Re-S/A). Quantitative PCR (qPCR) was used to quantify the virus load of RoWV-1. An absolute standard curve was constructed from a plasmid clone of the corresponding RoWV-1 genome region using specific primers ABVA/ABVS (Supplementary Table S1). PCR products were cloned into pGEM-T Easy vectors and then sequenced. Standard curves were generated by determination of copy numbers (10^2^–10^9^ copies) of standard plasmid. qPCR was performed using the CFX 96 Real-Time Detection System (Bio-Rad, Hercules, CA, USA) with tSYBR *Premix Ex* Taq II (Tli RNaseH Plus; Takara Bio, Otsu, Japan). Thermal cycling conditions were 94 °C for 30 s, 40 cycles of 95 °C for 5 s, and 60 °C for 30 s. Three biological replicates for each group were performed. The equation of *y* = −0.2482*x* + 12.227 (*y* = the logarithm of plasmid copy number to base 10; *x* = Ct value; *R*^2^ = 0.99) was used to calculate the copy number of RoWV-1 genomes.

### Polyclonal antibody preparation

A cDNA fragment of RoWV-1 capsid protein CP3 was amplified using primers VP3A and VP3S (Supplementary Table S1). The purified PCR product was cloned into vector pET-28a (+) (Novagen, CA, USA) with a His-tag and sequence was confirmed by DNA sequencing. The recombinant vector was transformed into BL21 (DE3) *Escherichia coli* bacterial strain (TransGen Biotech) and isopropyl β-D-1-thiogalactopyranoside (Sangon Biotech, Shanghai, China) was added to induce protein expression for 24 h. Bacterial cells were collected by centrifugation and disrupted with BugBuster Master Mix (Novagen, San Diego, CA, USA) using standard procedures. The insoluble recombinant His-tagged CP3-derived protein (31.5 kDa) was purified using Nichelating affinity columns (TransGen Biotech) under denaturing conditions. To confirm the identity of the recombinant protein, the protein was subjected to sodium dodecyl sulfate-polyacrylamide gel electrophoresis, transferred to a polyvinylidene difluoride membrane (Sigma, St. Louis, MO, USA) by a semi-dry electrophoretic transfer system (Bio-Rad), and detected with an anti-His monoclonal antibody conjugated to horseradish peroxidase (Hangzhou HuaAn Biotechnology, Hangzhou, Zhejiang Province, China). Signals were visualized with an enhanced chemiluminescence detection system (Super Signal West Pico Chemiluminescent Substrate; Pierce, Rockford, IL, USA). The recombinant plasmid was submitted to Wuhan Daian Biotechnology Company (Wuhan, Hubei Province, China) for production of purified protein, which was then used as an antigen for immunization of male New Zealand rabbits. The obtained polyclonal antibody to RoWV-1-CP3 protein was purified from New Zealand rabbit antiserum by the company. The company reported an antibody titer of 1:2000 and labeled with rabbit anti-RoWV-1-CP3.

### Determination of RoWV-1 tissue distribution and developmental expression patterns

Larvae, pupae, and adult wasps were collected to detect changes of RoWV-1 loads in *P. vindemmiae* wasps in different development stages. For the adult stage, females and males were collected daily from Day 1 (D1) to D7 after eclosion. Total RNA of each sample was extracted using TRIzol. The day with the highest virus load in the adult stage was selected to detect the distribution of RoWV-1 in different tissues. The specific steps were as follows: (1) a total of 30 infected females and 30 infected males were fed with a 10% honey solution and placed into two sterilized glass tubes, keeping females and males separated. (2) The tubes were placed on ice for 5–10 min. After chilling, wasps were placed in a 1:100 diluted solution of ribonuclease inhibitor (TransGen Biotech) and dissected under a dissecting microscope (Leica, Wetzlar, Germany). (3) Under the microscope, anatomical tweezers were used to remove each wasp’s head, thorax, and abdomen and to place them directly into TRIzol. (4) The digestive tracts, ovaries, venom glands, fat bodies, and testes were removed and placed directly into TRIzol. All wasp tissues or organs were dissected as one biological repeat; each experiment was repeated three times.

Similarly, the day with the highest virus load in larvae (D8) was selected for detecting the distribution of RoWV-1 in larvae. Under the microscope, digestive tracts, salivary glands, and fat bodies were dissected as described above.

*D. melanogaster* larvae, pupae, and adults were collected. For the adult stage, females and males were collected daily from D1 to D10 after eclosion from the puparia. The sample processing method and the tissue distribution detection in flies followed the same protocols described above for wasps.

### RoWV-1 antigen detection by immunohistochemistry

For immunohistochemistry (IHC), ovaries, testes, and digestive tracts from RoWV-1 (–) or RoWV-1 (+) insects were dissected, washed, and handled, as described previously [35]. The primary antibody was rabbit anti-RoWV-1-CP3, diluted 1:500 in 0.1 M, pH 7.0 phosphate-buffered saline (PBS; Wisent Biotech, Nanjing, Jiangsu Province, China), containing 0.3% Triton X-100 (Solarbio, Beijing, China) and 10% goat serum (Sangon Biotech). The secondary antibody was DyLight 549-conjugated goat anti-rabbit (Abbkine, Redlands, CA, USA), diluted 1:200 in 0.1 M, pH 7.0 PBS (Wisent Biotech, Nanjing, Jiangsu Province, China), containing 0.3% Triton X-100 (Solarbio, Beijing, China) and 10% goat serum (Sangon Biotech). Cytoskeletal actin was stained with Phalloidin-iFluor 488 Reagent, diluted 1:1000 in 0.1 M PBS, containing 0.3% Triton X-100. Cell nuclei were stained with 1 μg/mL 4′,6-diamidino-2-phenylindole (YEASEN Biotech, Shanghai, China). Samples were analyzed and images recorded using a LSM 780 confocal microscope (Carl Zeiss SAS, Jena, Germany). A stack of consecutive confocal optical sections (Z-stacks) was recorded at 8-bit resolution. Images were merged and scale bars were added using LSM ZEN 2010 software (Carl Zeiss SAS). Adobe Photoshop CC and Adobe Illustrator CC (Adobe Systems, San Jose, CA, USA) were used for image grouping. All samples were analyzed with the same microscope and software settings.

### Visualization of RoWV-1 particles by transmission electron microscopy (TEM)

Samples were prepared as described previously [31]. Briefly, the digestive systems of *P. vindemmiae* wasp larvae and the digestive tracts, ovaries, and testes of *D. melanogaster* were dissected under a dissecting microscope (Leica). The dissected samples were pre-fixed overnight at 4 °C with 2.5% glutaraldehyde (Electron Microscopy Sciences, Hatfield, PA, USA) in PBS (0.1 M, pH 7.0). After three washes in PBS for 15 min each, the samples were fixed with 1% osmium tetroxide (OsO_4_; Electron Microscopy Sciences) in PBS for 1–2 h and washed again three times. The samples were dehydrated by a graded series of ethanol (30%, 50%, 70%, 80%, 90%, 95%, and 100% for about 15–20 min each) and transferred to absolute acetone for 20 min. Samples were infiltrated with a 1:1 mixture of absolute acetone and the final Spurr’s resin (SPI Supplies, West Chester, PA, USA) mixture for 1 h at room temperature, transferred to a 1:3 mixture of absolute acetone and the final resin mixture for 3 h, and then placed into a final Spurr’s resin mixture overnight. Finally, each specimen was placed in an Eppendorf tube containing Spurr’s resin, incubated at 70 °C for >9 h, and then sectioned using an EM UC7 ultramicrotome (Leica). Ultrathin sections were double-stained with uranyl acetate (SPI Supplies, West Chester, PA, USA) for 5 min and alkaline lead citrate (Electron Microscopy Sciences) for 10 min and then were observed using an H-7650 TEM (Hitachi, Tokyo, Japan) at an accelerating voltage of 80 kV.

### Determination of vertical and horizontal transmission of RoWV-1

For the vertical transmission experiment, newly emerged *P. vindemmiae* wasps from the RoWV-1 (–) and RoWV-1 (+) colonies were individually kept in sterilized glass tubes and fed with a 10% honey solution for 24 h. For each crossing experiment, ten pairs of females and males were allowed to mate for 24 h. Both females and males were then individually moved to new sterilized glass tubes and provided 20 newly pupated *D. melanogaster* pupae for the female wasps to parasitize. To avoid offspring mating, wasp pupae were removed from the parasitized *D. melanogaster* pupae and individually kept in sterilized glass tubes until emergence. Once emerged, all females or males from the same parasitoid wasp parent were collected separately for RoWV-1 detection, using the methods described below. Four crossed experiments were designed: female (+) × male (+), female (–) × male (–), female (+) × male (–), and female (–) × male (+), in which (+) means infected with RoWV-1 and (–) means not infected with RoWV-1.

For horizontal transmission experiments, 60 newly emerged wasps from the RoWV-1 (+) *P. vindemmiae* wasp colonies were kept together in a sterilized glass tubes and fed with a 10% honey solution for 3 days. Subsequently, all wasps were moved to another new glass tube. Then, 60 newly emerged female wasps from the RoWV-1 (–) colony were placed into the contaminated tube in which the RoWV-1 (+) wasps had been held and fed. The same method was applied to the male wasps. Every 3 days, 15 wasps were collected from the tubes and tested for RoWV-1. Notably, in this experiment, all sponge plugs were cleaned to avoid contamination.

In the case of *D. melanogaster*, 30 RoWV-1-infected flies were pooled and ground in 600 µL PBS using two 3-mm glass beads in a TyssueLyser II (Qiagen, Hilden, Germany) at 25 Hz for 90 s. Debris was removed by centrifugation at 17,000 × *g* for 10 min, and supernatant was filter-sterilized to remove bacteria using a Millex GV 22 mm filter (Merck Millipore, Billerica, MA, USA). Following filtration, the collected suspension was spread in tubes containing standard cornmeal medium. Then, uninfected adult (age 4–7 days) flies were transferred to sterilized glass tubes containing fresh standard cornmeal medium. The next morning, 100 eggs were collected, placed on wet sterile filter paper (General Electric Biotechnology, Hangzhou, Zhejiang Province, China), and transferred into the tubes containing RoWV-1 virus extracts. Hatched larvae were maintained on cornmeal medium until collection. In a separate experiment, 30 infected *D. melanogaster* were placed into sterilized glass tubes, and 15 uninfected flies with red-labeled wings were placed into the same tubes. Three to five labeled flies were pooled every 2 days and homogenized to detect RoWV-1.

For vertical transmission experiments, newly emerged *D. melanogaster* from the RoWV-1 (–) and RoWV-1 (+) colonies were individually kept in sterilized glass tubes containing fresh food for 24 h. For each crossing experiment, ten pairs of females and males were allowed to mate for 24 h. Both females and males were then individually moved to new sterilized glass tubes containing fresh food to lay eggs. To avoid RoWV-1 contamination of food by parental *D. melanogaster*, eggs were collected next morning and transferred into tubes containing fresh food. Hatched larvae, pupae, and adult flies were collected separately for RoWV-1 detection. To avoid mating of adult flies, late-stage pupae were individually kept in sterilized glass tubes until emergence. Once emerged, females or males from the same parents were collected separately for RoWV-1 detection. Four crossed experiments were designed: female (+) × male (+), female (–) × male (–), female (+) × male (–), and female (–) × male (+), in which (+) means infected with RoWV-1 and (–) means not infected with RoWV-1.

### Determination of the effect of RoWV-1 infection on *P. vindemmiae* wasps

Major biological parameters, including degree of infestation, success rate of parasitism, offspring number per parasitized pupa, offspring sex ratio, developmental duration, adult longevity, mature eggs number and ovarioles number of *P. vindemmiae* wasps, were compared between the RoWV-1 (–) and RoWV-1 (+) colonies. The experiment was conducted as follows: a total of 30 female and 30 male newly emerged wasps were paired, and each pair was placed into a clean plastic or glass tube. Each pair was fed with a 10% honey solution and allowed to mate for 48 h. After mating, 20 *D. melanogaster* pupae (age 2 days) were placed into each tube (a ratio of 1:20 female wasps: fly pupae). Every 24 h, *D. melanogaster* pupae were individually transferred into new sterilized glass tubes, and fresh pupae were supplied. Fresh *D. melanogaster* pupae were replaced until the female wasp died, and collected fly pupae were kept until all wasps emerged.

After wasp offspring emerged, both female and male offspring from each tube were collected, and fly pupae that had not produced wasps were removed. The state and number of wasps that had not completed development were noted. Total numbers of adult *D. melanogaster* (*di*) and emerged wasps (*p*_*i*_) were documented. The degree of infestation (%DI) measures the proportion of hosts that were successfully parasitized and was estimated as ((*T* – *di*) / *T*) × 100; *T* is the average number of emerging flies in the absence of a wasp. The success rate of parasitism (%SP) measures the probability that an infested host will give rise to an adult wasp and was estimated as [*p*_*i*_ / (*T* – *di*)] × 100. In some cases, *p*_*i*_ was greater than (*T* – *di*); in these cases we set SP as 1 [25]. The biological significance of both parameters is clear: the degree of infestation represents the probability of a given host being parasitized, and the success rate of parasitism indicates the probability that a parasitized host would give rise to a wasp. The other biological parameters were calculated as follows: egg number per parasitized pupa = number of wasp eggs from each parasitized pupa; and offspring sex ratio (%) = total number of female adult wasps emerged from the same female wasp parasitized pupa/ total number of female and male adult wasps emerged from the same female wasp parasitized pupa × 100 [31].

### Determination of the effect of RoWV-1 infection on *D. melanogaster*

Major biological parameters, including fecundity, developmental duration, longevity of adult *D. melanogaster*, pupation rate, and eclosion rate, were compared between the RoWV-1 (–) and RoWV-1 (+) *D. melanogaster* colonies.

To determine *D. melanogaster* fecundity, newly emerged males and females (not mated) were collected separately. After 3 days, ten male flies and ten female flies were placed together into a tube to mate for 24 h and then into a fecundity device (Supplementary Fig. S1) for spawning [36]. Fecundity was not measured in the first 36 h because of incomplete mating. After 36 h, fecundity was measured every 12 h for 17 days, because short-term measurement of reproduction is well correlated with lifetime reproduction in *D. melanogaster* [37]. Three biological replicates were measured for each colony.

The other method used to measure *D. melanogaster* fecundity was described previously [38]. In brief, newly emerged males and females were placed together and allowed to mate for 3 days. Flies were then placed into 20 sterilized glass tubes (one male and one female per tube) containing fresh standard cornmeal medium to lay eggs. The paired flies were moved to new tubes with fresh media every 5 days until Day 30 post experiment. The number of emerged offspring from each individual female fly was recorded.

Longevity of *D. melanogaster* was measured by collecting newly emerged flies from and keeping them separately in sterilized glass tubes containing fresh standard cornmeal medium. Media were replaced every 5 days. Number of deaths were recorded daily until all flies had died naturally.

Developmental time was measured as previously described [39]. Briefly, females (*n* = 10) that had been with males (*n* = 10) for at least 2 days were placed into a large sterilized plastic tube (Hongtai experimental equipment) (18 × 82 mm) with axenic standard cornmeal medium and removed 4 h later. After pupation and adult emergence, the numbers of pupae and flies were documented twice a day (morning and evening). The offspring females and males (*n* = 90 for each) were counted.

The pupation rate and eclosion rate were measured as follows: 30 fully mated RoWV-1 (–) *D. melanogaster* females were collected and placed for 12 h into a fecundity device (Supplementary Fig. S1) that contained a grape juice medium to allow spawning. Fifty eggs were gently collected with a pen brush (2.5 × 11 mm) and placed on axenic standard cornmeal medium containing either isopycnic PBS or RoWV-1 virus extract. Media were then kept at 25 °C with 60 ± 5% relative humidity and a photoperiod of 16 h of light to 8 h of darkness for fly development. Finally, the number of successful pupae from the media and the number of successfully emerged adults were counted. Three biological replicates were measured for each treatment. The pupation rate was calculated as the number of successful pupae divided by the number of initial eggs (50 eggs). Due to technical limitations, we used eggs instead of larvae in the pupation rate calculation. The eclosion rate was calculated as the number of successfully emerged adults divided by the number of successful pupae.

### Statistical analysis

All values were expressed as mean ± standard error of mean. Infectious viral titers were compared by analysis of variance (ANOVA) after log_10_ (*X* + 1) transformed, but untransformed data are presented. All data were analyzed using a one-way ANOVA or two-way ANOVA, followed by Tukey’s multiple comparison test (*p* < 0.05). The mean of each measured biological parameter was compared between two colonies infected and uninfected by RoWV-1 using Student’s *t*-test (*p < 0.05; **p < 0.01; ***p < 0.001). All statistical calculations were performed using Data Processing System software (version 14.50) [40].

## Results

### *P. vindemmiae* wasps harbor a novel virus with a genome containing two ORFs, encoding four conserved domains and four capsid proteins

**Fig. 1:**
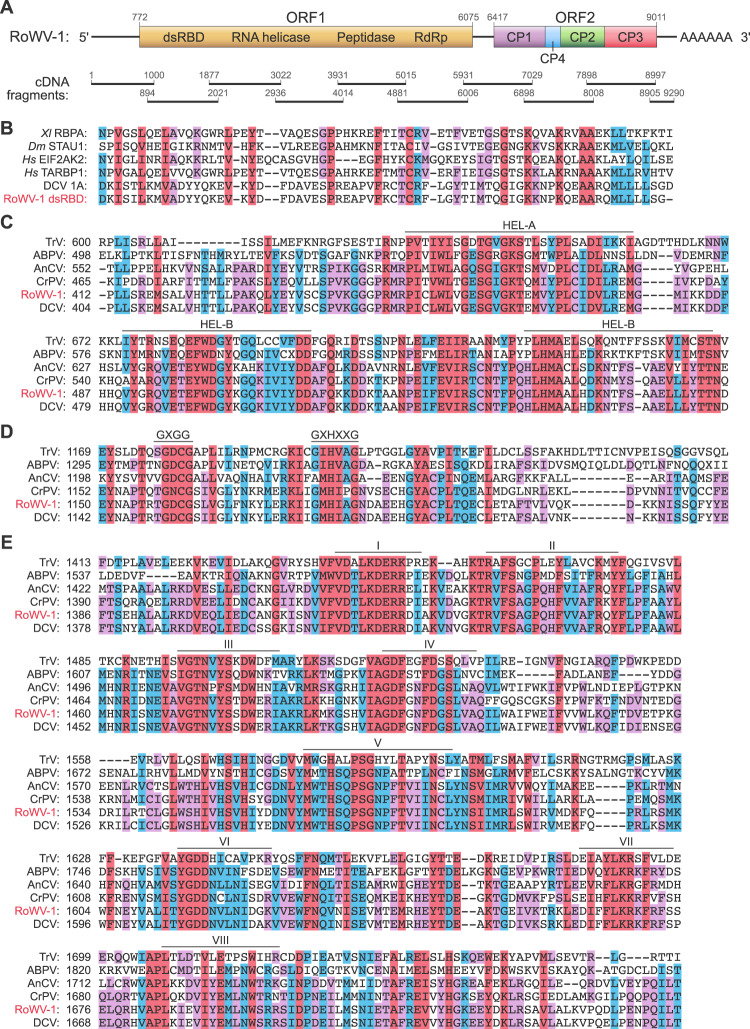
*Pachycrepoideus vindemmiae* wasps harbor a novel virus with a genome containing two open reading frames (ORFs), encoding four conserved domains and four capsid proteins. **A** Genomic organization of Rondani’s wasp virus 1 (RoWV-1). Numbers indicate nucleotide positions, and boxes represent ORFs. The positions of structural proteins (capsid proteins C1–4) and nonstructural proteins (dsRBD, double-strand RNA-binding domain; RNA helicase; peptidase; RdRp, RNA-directed RNA polymerase) are indicated according to scale, as are cDNA fragments used for viral genome sequencing. **B** Alignment of the putative dsRBD of RoWV-1 (aa 31–98) and dsRBDs of proteins from different model organisms and the dsRBD of drosophila C virus (DCV) 1A protein. *Xl* RBPA: *Xenopus laevis* RNA-binding protein A (*Xl* RBPA2); *Dm*STAU1: *Drosophila melanogaster* staufen double-strand RNA-binding protein 1; *Hs* EIF2AK2: *Homo sapiens* eukaryotic translation initiation factor 2 alpha kinase 2; *Hs* TARBP1: *Homo sapiens* TAR (HIV-1) RNA-binding protein 1. Names and abbreviations of dsRBD homologs encoded by various organisms and associated protein accession numbers are listed in Supplementary Table S4. **C** Alignment of the conserved regions of the putative RNA helicase of RoWV-1 with RNA helicases of Triatoma virus (TRV), acute bee paralysis virus (ABPV), anopheles C virus (AnCV), cricket paralysis virus (CrPV), and DCV. Conserved motifs are indicated by labeled dashes. **D** Alignment of the putative peptidase RoWV-1 with the peptidases those of other viruses. **E** Alignment of the putative RdRp of RoWV-1 with those of other viruses. Conserved motifs are indicated by full lines and Roman numerals. Numbers on the left in **C**–**E** show amino acid positions. Protein sequence accession numbers are listed in Supplementary Table S2.

Transcriptomic data of *P. vindemmiae* wasps (Supplementary Table S3) were obtained by Illumina sequencing and assembled using Trinity v2012-10-05 without a reference genome. Analysis of the assembled transcriptome of *P. vindemmiae* wasps uncovered a 9312-nucleotide-long contig, containing regions like those of viral RdRps. Consequently, we determined the sequence of associated viral genome, including the 5’ and 3’ genome termini, by RACE. The complete genome of the novel virus, here named RoWV-1, is 9332 nucleotides in length and contains a polyadenylate tail at the 3’ end. G+C pairs comprise 39.9% of the nucleotides (28.7% A, 18.4% C, 21.5% G, and 31.4% U). The RoWV-1 genome contains two non-overlapping ORFs (ORF1 and ORF2) in a linear arrangement. ORF1 (genome nucleotides 772–6075) and ORF2 (genome nucleotides 6417–9011) encode proteins of 1768 and 865 amino acid residues, respectively (Fig. 1A).

Analysis of the four domains yielded the following information: dsRBD consists of 68 amino acid residues and aligns with dsRBDs of, for instance, Drosophila C virus (DCV; *Dicistroviridae*: *Cripavirus*) and cellular proteins from different model organisms (Supplementary Table S4) via highly conserved amino acid residues (Fig. 1B). The RNA helicase domain is 175 amino acid residues long and contains three canonical helicase motifs: A, B, and C [41] (Fig. 1C). The peptidase domain (comprised of 146 amino acid residues) is highly similar to picornaviral 3C proteases [41] and contains two conserved motifs (Fig. 1D). The RdRp domain (comprised of 396 amino acid residues) contains eight conserved motifs, with highest similarity to those found in dicistrovirus RdRps (Fig. 1E). Alignment by ClustalW clearly indicated that the RoWV-1 RdRp is most closely related to that of DCV. Together, these results indicate that RoWV-1 is a novel member of the order *Picornavirales*. It is unlikely that RoWV-1 is a contaminant because RoWV-1 could be detected in populations of *P. vindemmiae* wasps raised in a laboratory located in Anhui Province (Supplementary Fig. S2A) but not in the wild populations of *D. melanogaster* collected in different provinces of China (Supplementary Table S5).

### RoWV-1 is a novel cripavirus

**Fig. 2:**
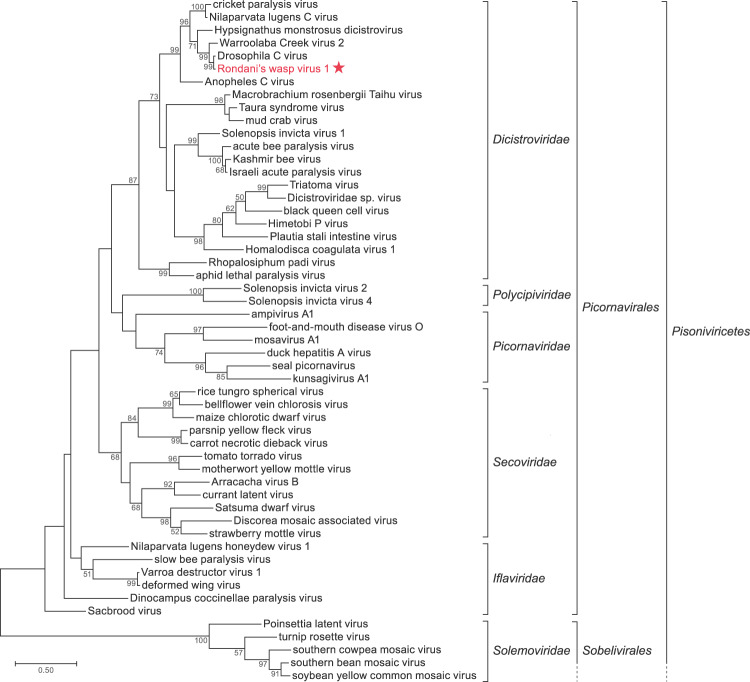
Rondani’s wasp virus 1 (RoWV-1) is a novel cripavirus. Phylogenetic tree of the RdRp domain of RoWV-1 and selected positive-sense RNA viruses. RoWV-1 is indicated by a red star. Bootstrap values represent 1000 replicates. Virus names and protein accession numbers are listed in Supplementary Table S2.

Using the deduced amino acid sequence of the most conserved viral domain, RdRp, we investigated the possible phylogenetic relationship of RoWV-1 and other picornavirals. The resulting phylogenetic tree indicated that RoWV-1 is a novel member of dicistrovirid genus *Cripavirus*, most closely related to DCV (Fig. 2). PASC analysis revealed the RoWV-1 genome to be 81.4% identical to the genome of DCV, 53.6% identical to that of CrPV, 44.0% identical to that of Anopheles C virus (*Dicistroviridae*, likely a cripavirus), and 29.2% identical to that of acute bee paralysis virus (*Dicistroviridae*: *Aparavirus*). These results indicated that RoWV-1 represents a novel cripavirus species.

### RoWV-1 load is highest in larval *P. vindemmiae* wasp digestive tracts

**Fig. 3:**
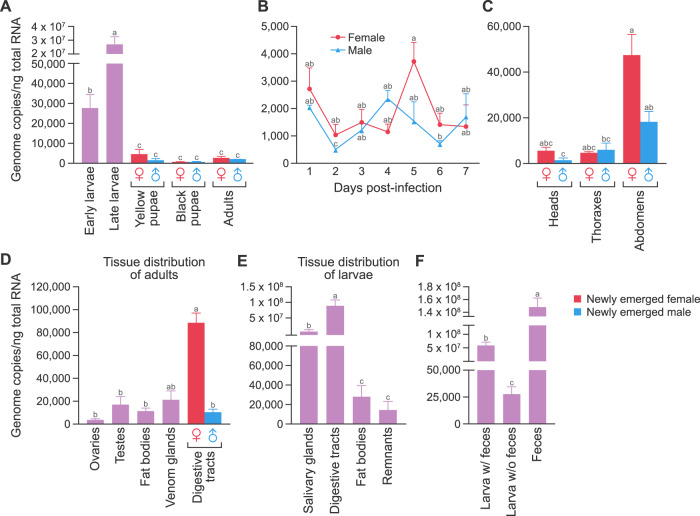
Rondani’s wasp virus 1 (RoWV-1) load is highest in larval *Pachycrepoideus vindemmiae* wasp digestive tracts. **A** RoWV-1 load in larvae, pupae, and adult wasps. Adults refer to newly emerged female and male adults. **B** RoWV-1 load in adult wasps at Day 1 through Day 7 after eclosion. **C** RoWV-1 load in heads, thoraxes, and abdomens. **D** RoWV-1 load in adult ovaries, testes, fat bodies, female digestive tracts, male digestive tracts, and venom glands. **E** RoWV-1 load in larval salivary glands, digestive tracts, fat bodies, and remnants. **F** RoWV-1 load in late larvae with/without feces and late larval feces. Data represent means ± standard error of mean (SEM). Bars annotated with identical letters do not differ significantly (Tukey’s multiple comparison test). Multiple comparisons in one-way or two-way ANOVA are shown with lowercase letters, showing statistically significant difference at *p* < 0.05 for each treatment. A completely different letter means there is a significant difference between the two (e.g., a and b), but when the same letter is displayed, there is no significant difference between the two (e.g., a and a or a and ab).

Developmental expression analysis detected RoWV-1 in early larvae, late larvae, pupae, and adults of *P. vindemmiae* wasps. Virus load, as determined by vRoWV-1 genome copy number, increased significantly along with larval development ANOVA results: F statistic = 60.822; degrees of freedom [DF] = 7; *p* value < 0.001 [Fig. 3A]) and peaked in late larvae. However, genome copy number declined to basal level in the pupal stage (Fig. 3A) and was not significantly different in female and male adults (F = 1.33, DF = 1, *p* = 0.2586 [Fig. 3B]), and viral loads in D2 and D5 were significantly different (F = 3.3670, DF = 6, *p* = 0.0126 [Fig. 3B]).

RoWV-1 virus loads in heads, thoraxes, and abdomens of adult *P. vindemmiae* wasps were significantly different (F = 14.909, DF = 2, *p* < 0.001 [Fig. 3C]), and female abdomens contained significantly more viral RNA than male abdomens (F = 6.064, DF = 1, *p* = 0.0299 [Fig. 3C]). Tissue-specific distribution analysis (F = 8.035, DF = 5, *p* = 0.0016 [Fig. 3D]) revealed the highest virus load in female digestive tracts, significantly higher compared to virus loads in other tissues. However, comparing testes tissues (or ovaries), fat bodies, venom, and male digestive tracts, there were no significant differences. The highest virus load in larval tissues was detected in digestive tracts (F = 30.993, DF = 3, *p* = 0.0015 [Fig. 3E]), a result similar to that of adult tissue analysis. Moreover, virus load decreased sharply after mature wasp larvae excreted digestive tract contents. At the same time, a high virus load was detected in larval feces (F = 556.04, DF = 2, *p* < 0.001 [Fig. 3F]).

### RoWV-1 is widely distributed in *P. vindemmiae* wasp tissues

**Fig. 4:**
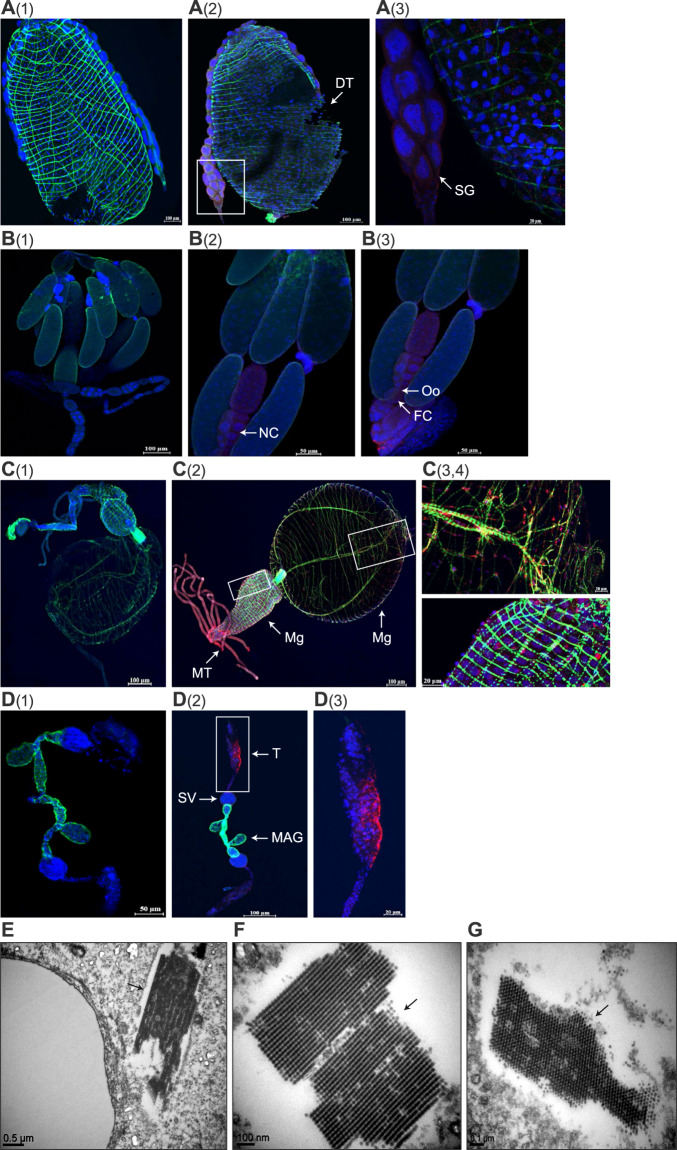
Rondani’s wasp virus 1 (RoWV-1) is widely distributed in *Pachycrepoideus vindemmiae* wasp tissues. **A** Localization of RoWV-1 antigen (red) in larval salivary gland and digestive tract (IHC). **A(1)**, **A(2)** Larval digestive tract and salivary gland, stains were derived from dissected RoWV-1 (–) and RoWV-1 (+) wasps, respectively. **A(3)** The enlarged insets of the boxes in **A(2)**. SG salivary gland, DT digestive tract. **B** Localization of RoWV-1 antigen (red) in ovary (IHC). FC follicle cell, NC nurse cell, Oo oocyte. **B(1)**, **B(2)**, **B(3)** Adult wasp’s ovary, stains were derived from dissected RoWV-1 (–) and RoWV-1 (+) wasps, respectively. **C** Localization of RoWV-1 antigen (red) in digestive tract (IHC). **C(1)**, **C(2)** Adult wasp’s digestive tract, stains were derived from dissected RoWV-1 (–) and RoWV-1 (+) wasps, respectively. **C(3)**, **C(4)** The enlarged insets of the boxes in C(2). Fg foregut, Mg midgut, MT Malpighian tubule. **D** Localization of RoWV-1 antigen (red) in testis (IHC). **D(1)**, **D(2)** Adult wasp’s testis, stains were derived from dissected RoWV-1 (–) and RoWV-1 (+) wasps, respectively. **D(3)** The enlarged inset of the box in **D(2)**. MAG male accessory gland, SV seminal vesicle, T testis. F-actin was stained with phalloidin (green). Cell nuclei were stained with DAPI (blue). **E**, **F** RoWV-1 particles observed in larval digestive tracts (arrows) on transmission electron micrographs. **G** RoWV-1 particles observed in larval feces (arrows) on transmission electron micrographs.

IHC revealed RoWV-1 antigen in larval salivary glands and digestive tracts (Fig. 4A(2), A(3)), adult ovaries (Fig. 4B(2), B(3)), foreguts, midguts (Fig. 4C(2)–C(4)), and testes (Fig. 4D(2), D(3)) of infected wasps, RoWV-1 (+). Interestingly, RoWV-1 antigen was present in nurse cells and follicular cells of immature follicles but not in terminal mature oocytes. Similarly, RoWV-1 antigen was detected only in testicular follicles but not in matured spermatozoa (sperm). RoWV-1 antigen could not be detected in tissues sampled from uninfected wasps, RoWV-1 (–) (Fig. 4A(1), B(1), C(1), D(1)).

To study the morphology of RoWV-1 particles, we prepared cross-sections of the digestive tract of RoWV-1 (+) larvae with high virus loads and examined them using TEM. Spherically shaped virion-like particles (VLPs), 28–30 nm in diameter (Supplementary Fig. S3), similar to those produced by other cripaviruses (e.g., DCV) [42], were present in numerous digestive tract cells (Fig. 4E, F). Similarly, VLPs were also observed in the feces of larvae (Fig. 4G). Most VLPs were arranged in paracrystalline structures similar to other cripavirus infections (e.g., DCV) [42]. As expected, VLPs were not found in RoWV-1 (–) wasp larvae. Together, these results indicate that RoWV-1 widely infects tissues of its wasp host.

### RoWV-1 is transmitted horizontally, but not vertically, by *P. vindemmiae* wasps

**Fig. 5:**
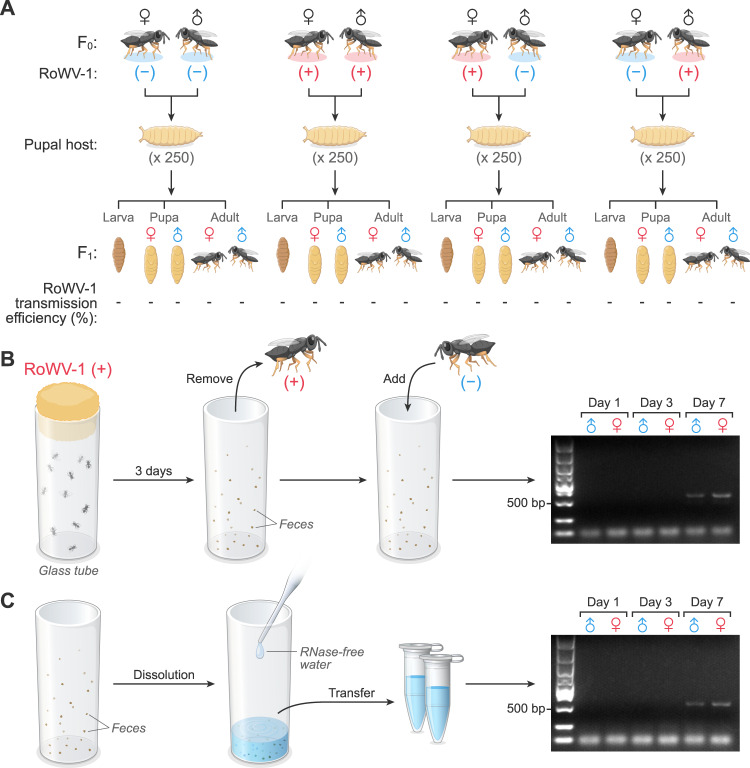
Rondani’s wasp virus 1 (RoWV-1) is transmitted horizontally, but not vertically, by *Pachycrepoideus vindemmiae* wasps. **A** Vertical RoWV-1 transmission to offspring (F1) and **B** horizontal RoWV-1 transmission from RoWV-1 (–) to RoWV-1 (+) wasps. RoWV-1 infection was determined by RT-PCR with specific primers PVDA-1/PVDS-1 and PVDA-2/PVDS-2 followed by gel electrophoresis at the indicated days. **C** RoWV-1 contamination in feces remaining tubes that had held wasps. Feces were dissolved in RNase-free water and RoWV-1 was detected as in **B**.

To examine the vertical transmission capability of RoWV-1, we collected the offspring of RoWV-1 (+) wasps at different developmental stages for viral genome detection by PCR. The results (Supplementary Fig. S4) indicate that RoWV-1 is not transmitted from infected parents to their offspring (Fig. 5A). This result is consistent with the absence of RoWV-1 in mature oocytes and sperm as judged by IHC (Fig. 4). On the other hand, PCR using two pairs of specific RoWV-1 primers indicated that RoWV-1 (–) wasps could be infected with RoWV-1 after their exposure to places previously in contact with RoWV-1 (+) wasps (Fig. 5B). Supporting this finding, RoWV-1 was detected in the wash solution used to clean tubes in which RoWV-1 (+) wasps had been held and in which they had defecated (Fig. 5C). Together, these results indicate that RoWV-1 can be transmitted from RoWV-1 (+) to RoWV-1 (–) wasps horizontally via contact with contaminated feces. RoWV-1 infection was determined by semi-qPCR.

### RoWV-1 has no obvious effects on *P. vindemmiae* wasps

**Fig. 6:**
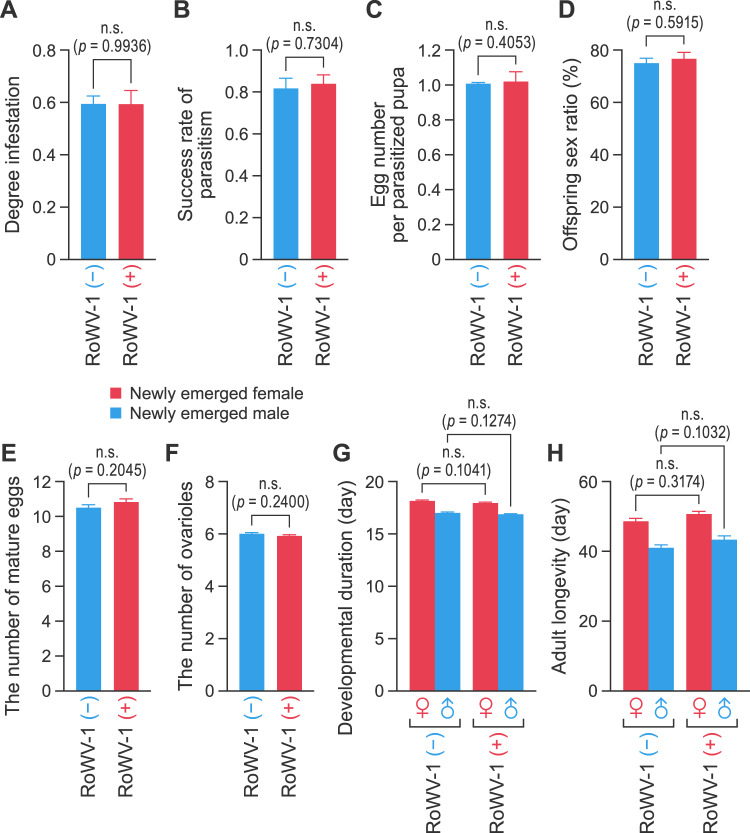
Rondani’s wasp virus 1 (RoWV-1) has no detrimental effects on *Pachycrepoideus vindemmiae* wasps. Comparison of RoWV-1 infected (+) and uninfected (–) *P. vindemmiae* wasps’ colonies regarding **A** degree of infestation (*n* = 30 independent samples), **B** success rate of parasitism (*n* = 30 independent samples), **C** egg number per parasitized pupa (*n* = 30 independent samples), **D** offspring sex ratio (*n* = 30 independent samples), **E** the number of mature eggs (*n* = 66 independent samples), **F** the number of ovarioles (*n* = 66 independent samples), **G** developmental duration (*n* = 30 independent samples), and **H** adult longevity (*n* = 129 independent samples for female and *n* = 69 independent samples for female). Data represent means ± standard error of mean (SEM). Statistical significance (*t-*test) is indicated by asterisks: *p < 0.05; **p < 0.01; ***p < 0.001.

To discover the effects of RoWV-1 on *P. vindemmiae* wasps, we compared some important biological parameters of infected versus uninfected wasps. Comparison of RoWV-1 (+) and RoWV-1 (–) wasp colonies via *t*-test revealed no significant differences in degree of infestation of *D. melanogaster* (*t* = 0.0080, DF = 58, *p* = 0.9936 (Fig. 6A]), success rate of parasitism (*t* = 0.3462, DF = 58, *p* = 0.7304 [Fig. 6B]), egg number per parasitized pupa (*t* = 0.8383, DF = 58, *p* = 0.4053 [Fig. 6C]), offspring sex ratio (*t* = 0.5397, DF = 58, *p* = 0.5915 [Fig. 6D]), the number of mature eggs (*t* = 1.2752, DF = 130, *p* = 0.2045 [Fig. 6E]), the number of ovarioles (*t* = 1.1806, DF = 130, *p* = 0.2400 [Fig. 6F]), developmental duration (female: *t* = 1.6514, DF = 58, *p* = 0.1041; male: *t* = 1.5467, DF = 58, *p* = 0.1274 [Fig. 6G]), and adult longevity (female: *t* = 1.9329, DF = 236, *p* = 0.3174; male: *t* = 1.6406, DF = 136, *p* = 0.1032 [Fig. 6H]). These results indicated that RoWV-1 infection has no significant impact on *P. vindemmiae* wasps.

### RoWV-1 is able to infect *D. melanogaster*

**Fig. 7:**
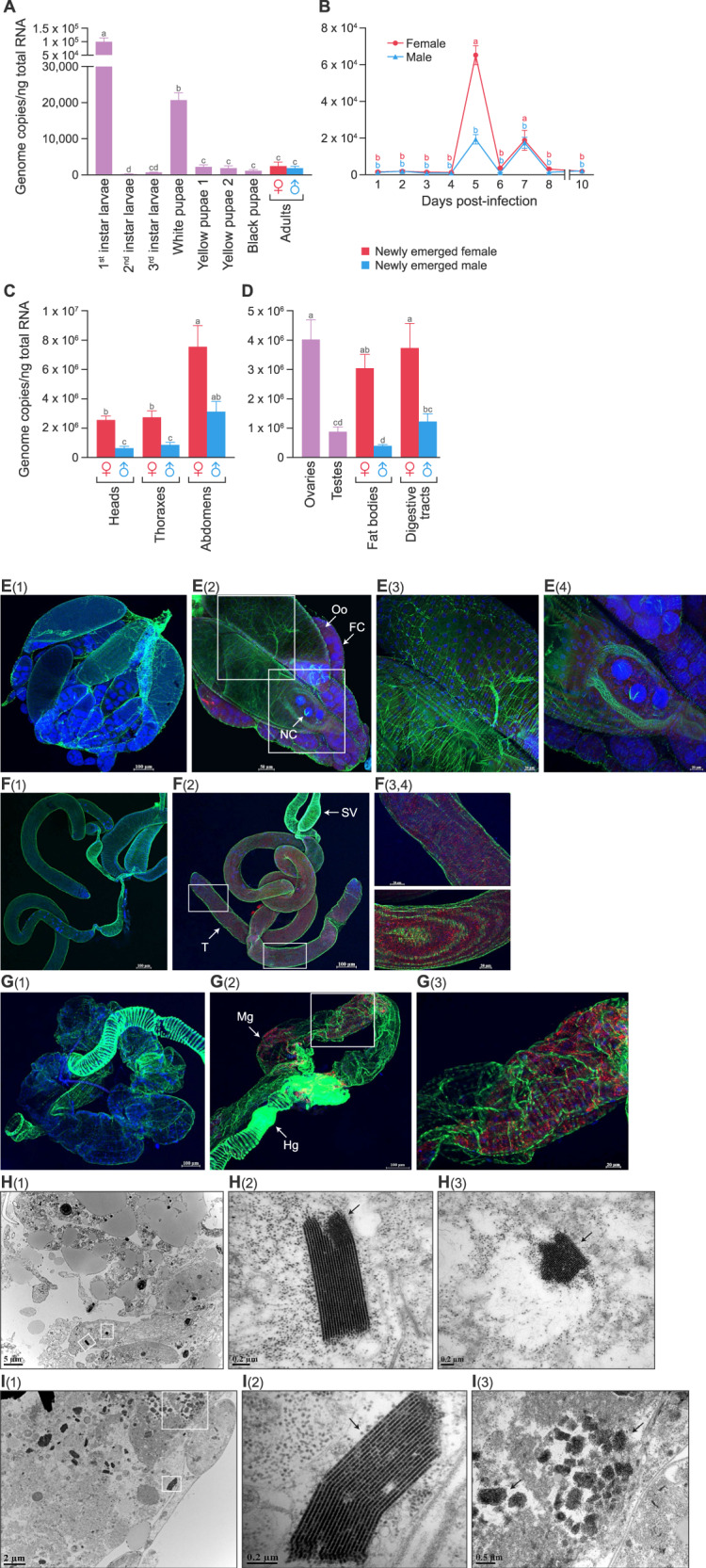
Rondani’s wasp virus 1 (RoWV-1) is able to infect *Drosophila melanogaster*. **A** RoWV-1 load in larvae, pupae, and adult *D. melanogaster*: yellow pupae 1, early-stage yellow pupae; yellow pupae 2, late-stage yellow pupae; adults refer to newly emerged male and female adults. **B** RoWV-1 load in adult flies at Day 1 through Day 10 after eclosion. **C** RoWV-1 load in *D. melanogaster* heads, thoraxes, and abdomens. **D** RoWV-1 load in adult *D. melanogaster* ovaries, testes, female digestive tracts, male digestive tracts, female fat bodies, and male fat bodies. Data represent means ± standard error of mean (SEM). Bars annotated with identical letters do not differ significantly (Tukey’s multiple comparison test). **E** Localization of RoWV-1 antigen (red) in *D. melanogaster* ovary. **E(1)**, **E(2)** Adult fly’s ovary, stains were derived from dissected RoWV-1 (–) and RoWV-1 (+) *D. melanogaster*. **E(3)**, **E(4)** The enlarged insets of the boxes in **E(2)**: FC follicle cell, NC nurse cell, Oo oocyte. **F** Localization of RoWV-1 antigen (red) in *D. melanogaster* testis. **F(1)**, **F(2)** Adult fly’s testis, stains were derived from dissected RoWV-1 (–) and RoWV-1 (+) *D. melanogaster*. **F(3)**, **F(4)** The enlarged insets of the boxes in **F(2)**. SV seminal vesicle, T testis. **G** Localization of RoWV-1 antigen (red) in *D. melanogaster* digestive tracts. **G(1)** Adult fly’s digestive tract, and **G(2)** adult fly’s digestive tract, stains were derived from dissected RoWV-1 (–) and RoWV-1 (+) *D. melanogaster*. **G(3)** The enlarged inset of the box in **G(2)**: Hg hindgut, Mg midgut. F-actin was stained with phalloidin (green). Cell nuclei were stained with DAPI (blue). **H** RoWV-1 particles observed in ovary (arrows) on transmission electron micrographs. **H(2)**, **H(3)** The enlarged insets of the boxes in **H(1)**. **I** RoWV-1 particles observed in testis (blue arrows) on transmission electron micrographs. **I(2)**, **I(3)** The enlarged insets of the box in **I(1)**. Multiple comparisons in one-way or two-way ANOVA are shown with lowercase letters, showing statistically significant difference at *p* < 0.05 for each treatment. A completely different letter means there is a significant difference between the two (e.g., a and b), but when the same letter is displayed, there is no significant difference between the two (e.g., a and a or a and ab).

We hypothesized that RoWV-1 also infected the hosts of *P. vindemmiae* wasps. To test this hypothesis, we exposed *D. melanogaster* to the crude RoWV-1 extract via abdominal injection. Developmental expression analysis detected a high virus load in the first instar larvae (F = 46.38, DF = 8, *p* < 0.001 [Fig. 7A]) but very low virus loads in larvae, pupae, and adults of *D. melanogaster*. In the adult stage, RoWV-1 virus load increased sharply on the fifth day after adult eclosion from the puparium (F = 62.72, DF = 8, *p* < 0.001 [Fig. 7B]) but then decreased and remained at a very low level. Female adults contained more virus than male adults (F = 15.152, DF = 1, *p* < 0.001[Fig. 7B]).

The virus loads in the heads, thoraxes, and abdomens of RoWV-1 (+) *D. melanogaster* were significantly different (F = 29.074, DF = 2, *p* < 0.001 [Fig. 7C]), and female abdomens contained significantly more viral RNA compared to male abdomens (F = 56.993, DF = 1, *p* < 0.001 [Fig. 7C]), a result similar to that observed in *P. vindemmiae* wasps (Fig. 3C). Specific tissue distribution analysis revealed that virus load was not significantly different between the different female tissues or between the different male tissues. However, all female tissues contained more viral RNA than male tissues (F = 21.537, DF = 5, *p* < 0.001 [Fig. 7D]).

IHC revealed RoWV-1 antigen in ovaries (Fig. 7E(2)–E(4)), testes (Fig. 7F(2)–F(4)), and midgut (Fig. 7G(2), G(3)) of the RoWV-1 (+) *D. melanogaster*. Similar to the results obtained for *P. vindemmiae* wasps (Fig. 4), RoWV-1 antigen was present in *D. melanogaster* nurse cells and follicular cells of immature follicles but not in the terminal mature oocytes, whereas RoWV-1 antigen was detected only in testicular follicles but not in matured spermatozoa (sperm). Unsurprisingly, RoWV-1 antigen could not be detected in tissues sampled from RoWV-1 (–) *D. melanogaster* (Fig. 7E(1), F(1), G(1)).

We also prepared sections of adult *D. melanogaster* ovaries and testes from RoWV-1 (+) colonies and examined them by TEM. Spherically shaped VLPs were present in ovarian cells (Fig. 7H(1)–H(3)). Similarly, VLPs were observed in the testes (Fig. 7I(1)–I(3)). As described for *P. vindemmiae* wasps, most VLPs were arranged in paracrystalline structures in immature gametes. As expected, VLPs were not found in RoWV-1 (–) *D. melanogaster*. Together, these results indicate that RoWV-1 widely infects tissues of at least one host of *P. vindemmiae* wasps.

### RoWV-1 is transmitted horizontally, but not vertically, by *D. melanogaster*

To examine the vertical transmission capability of RoWV-1, we collected the offspring of RoWV-1 (+) *D. melanogaster* at different developmental stages for viral genome detection by PCR (Supplementary Fig. S5). Similar to results obtained for RoWV-1 (+) wasps, we found that RoWV-1 is not transmitted from infected parents to their offspring (Supplementary Fig. S6A). But, RoWV-1 (–) *D. melanogaster* can become infected with RoWV-1 after exposure to RoWV-1 (+) flies (Supplementary Fig. S6B). When we transferred uninfected *D. melanogaster* eggs into sterilized glass tubes containing extracts of RoWV-1 (+) *D. melanogaster*, the hatched larvae, pupae, and emerged adult flies were RoWV-1 (+), as judged by PCR (Supplementary Fig. S6C). These results indicated that horizontal transmission of RoWV-1 among *D. melanogaster* is similar to that among *P. vindemmiae* wasps, despite these two insects belonging to two different taxonomic orders (Diptera versus Hymenoptera).

### RoWV-1 transmits bidirectionally between *P. vindemmiae* wasps and *D. melanogaster*

To determine the intricate transmission routes of RoWV-1 between *P. vindemmiae* wasps and *D. melanogaster* hosts, we collected *D. melanogaster* pupae after being parasitized by RoWV-1 (+) *P. vindemmiae* wasps. We found that RoWV-1 could be detected in RoWV-1 (–) *D. melanogaster* pupae after being parasitized by RoWV-1 (+) wasps (Supplementary Fig. S7A). More specifically, all of the *D. melanogaster* pupae were first confirmed to be parasitized by the wasps through examining the wasp eggs in the *D. melanogaster* pupae, and all the wasp eggs laid on the *D. melanogaster* pupae were then removed before RNA extraction prior to RT-PCR. Separately, we collected uninfected wasp eggs and then let them feed on infected *D. melanogaster* pupae. After 6 days, the wasp larvae were collected for RoWV-1 detection individually by RT-PCR. The results showed that the RoWV-1 (–) wasp larvae could be infected with RoWV-1 when feeding on the RoWV-1 (+) flies, although the infection rate was not 100% (Supplementary Fig. S7B). In addition, when uninfected wasps were allowed to parasitize the infected *D. melanogaster* pupae for 1 or 4 h, RoWV-1 could be detected (Supplementary Fig. S7C).

### RoWV-1 increases the developmental duration and fecundity of *D. melanogaster*

**Fig. 8:**
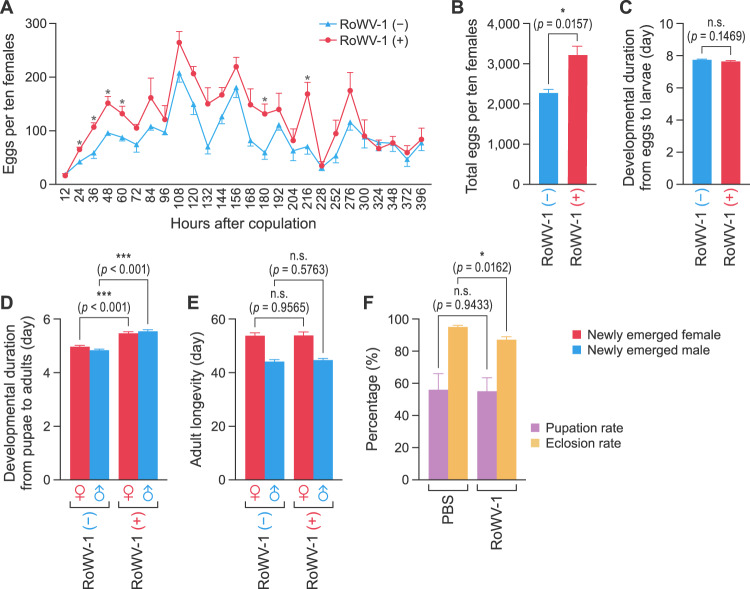
Rondani’s wasp virus 1 (RoWV-1) increases the developmental duration and fecundity of *Drosophila* melanogaster. Comparison of RoWV-1-infected (+) and uninfected (–) *D. melanogaster* colonies regarding **A** fecundity every 12 h, **B** total fecundity over 17 days (*n* = 3 independent samples), **C** developmental duration from eggs to larvae (*n* = 90 independent samples), **D** developmental duration from pupae to adults (*n* = 90 independent samples for females and *n* = 90 independent samples for males), **E** adult longevity (*n* = 85 independent samples for females and *n* = 90 independent samples for males), **F** pupation rate and eclosion rate (*n* = 3 independent samples). Data represent means ± standard error of mean (SEM). Statistical significance (*t-*test) is indicated by asterisks: *p < 0.05; **p < 0.01; ***p < 0.001.

Comparison of RoWV-1 (+) to RoWV-1 (–) *D. melanogaster* colonies via *t*-test revealed that RoWV-1 (+) *D. melanogaster* laid more eggs than their uninfected counterparts, as indicated by the total eggs per ten females every 12 h (Fig. 8A) or the total number of eggs laid per ten females over 17 days (*t* = 4.0317, DF = 4, *p* = 0.0157 [Fig. 8B]). There were no significant differences in developmental duration from eggs to pupae (*t* = 1.4568, DF = 178, *p* = 0.1469 [Fig. 8C]). However, RoWV-1 (+) *D. melanogaster* required more development time from the pupal to the adult stage (female: *t* = 6.3733, DF = 178, *p* < 0.001; male: *t* = 9.5062, DF = 178, *p* < 0.001 [Fig. 8D]), but adult longevity was not significantly affected (female: *t* = 0.0546, DF = 168, *p* = 0.9565; male: *t* = 0.5598, DF = 178, *p* = 0.5763 [Fig. 8E]). The pupation rate was not significantly different (*t* = 0.0757, DF = 4, *p* = 0.9433, [Fig. 8F]), whereas the eclosion rate decreased by 10% after flies were exposed to RoWV-1 virus extract (*t* = 3.995, DF = 4, *p* = 0.0162 [Fig. 8F]).

## Discussion

Most known RNA viruses encode an RdRp to replicate their genomes. Typically, these RdRps act in combination with other viral and host factors [43]. High error rates (typically on the order of 10^–4^) of RdRps ensure wide variability in RNA virus populations, allowing rapid viral evolution under selective pressures [44]. Generally, the RdRp is used as a reliable protein for constructing phylogenetic trees for the classification of RNA viruses, as it tends to be highly conserved among related groups [45, 46]. Phylogenetic analysis of the RoWV-1 RdRp revealed RoWV-1 to be a member of family *Dicistroviridae*, an emerging family of a positive-sense RNA viruses in the order *Picornavirales*. Dicistrovirids, including RoWV-1, are characterized by having genomes with two non-overlapping ORFs that encode nonstructural (5’-ORF) and structural proteins (3’-ORF) that mediate replication/polyprotein processing and capsid formation, respectively. An intergenic region, which functions as an internal ribosome entry site (IRES), separates both ORFs [47]. Phylogenetically, RoWV-1 closely clusters with dicistrovirid cripaviruses, in particular DCV, which commonly infects *D. melanogaster*. ORF1 and ORF2 of these two viruses share 92.3% and 77.9% amino acid sequence identity, respectively. The amino acid sequence identity of the capsid proteins of the two viruses is 77.9%. According to the International Committee on Taxonomy of Viruses demarcation criteria for genus *Cripavirus* (10% divergence threshold in the capsid protein sequence; https://talk.ictvonline.org/ictv-reports/ictv_online_report/positive-sense-rna-viruses/w/dicistroviridae/560/genus-cripavirus) and additional phylogenetic analyses of RoWV-1 RdRp domain, capsid protein, and complete genome, using other cripaviruses and more distantly related dicistrovirids (Supplementary Fig. S8), we consider RoWV-1 a representative of a new cripavirus species.

We found RoWV-1 in all female and male *P. vindemmiae* wasp tissues. The highest virus load was measured in the digestive system of wasp larvae. With the growth of larvae, virus load increased gradually, reaching a peak in mature larvae, and then decreased sharply in the pupal stage. Simultaneously, we also detected very high virus loads in larval feces (Fig. 3F). The sharp decrease in pupae, accompanied by the high virus load in larval feces, suggests that RoWV-1 is discharged from wasp feces in large quantities, which may be a primary spreading mechanism for RoWV-1.

Dicistrovirid replication occurs exclusively in the cytoplasm of infected cells [48]. TEM examination revealed the presence of RoWV-1 in the cytoplasm of infected cells either organized in crystalline structures or in an unarranged order. These differences in dispersal of the virus may reflect various stages of viral infection. The accumulation of viral particles in large crystalline lattices (Fig. 4E, F) and the dispersal of numerous particles in the cytoplasm (Fig. 4A) indicate that RoWV-1 replicates in the wasps. Large viral crystalline structure was found in the digestive tracts and feces of wasp larva, suggesting that RoWV-1 could accumulate in large quantities in the late larvae and be expelled via defecation. The virus forming a crystalline structure is probably a method of self-protection because of the very complex external environment.

Dicistrovirids may be transmitted horizontally per os from females to males [49] and vertically by the transovum [50] or transovarial route [51, 52]. However, DCV does not transmit vertically through the ova but rather horizontally [53]; DCV can be transmitted when *D. melanogaster* are in contact with contaminated *D. melanogaster* feces or cadavers [54]. Similarly, RoWV-1 does not transmit vertically but can be transmitted horizontally from infected wasps to uninfected wasps (Fig. 5B) via feces (Fig. 5C). Our study also demonstrated that RoWV-1 does not have obvious detrimental effects on *P. vindemmiae* wasps.

Parasitoid wasp larvae or pupae can acquire host insect viruses that subsequently evolve to benefit the wasps[55, 56]. Parasitoid wasp viruses can also infect the wasps’ hosts, resulting in biological and physiological constellations that may benefit the wasps [57–61]. Therefore, we investigated whether RoWV-1 could also infect and affect *D. melanogaster*, a host of *P. vindemmiae* wasps. Indeed, RoWV-1 could be detected in *D. melanogaster*, with the highest virus loads measured in the first instar larvae. Although RoWV-1 particles could be detected in the immature follicle cells of the reproductive systems of both parasites and hosts, they were not present in the terminal mature gametes, supporting our conclusion that RoWV-1 could not be transmitted vertically in either insect. However, RoWV-1 is able to transmit from *P. vindemmiae* to *D. melanogaster* pupae during parasitism, and RoWV-1 can also transmit from *D. melanogaster* to *P. vindemmiae* offspring and parents.

DCV infection increases ovariole number, daily egg production [62], and fertility [63], and decreases developmental time in *D. melanogaster* [39]. Sub‑lethal DCV infection results in a significant increase in reproduction, but this effect depended on host genotype [38]. Because of the high genomic similarity of DCV and RoWV-1, we hypothesized that RoWV-1 infection has similar effects. Our results further demonstrated that the infected *D. melanogaster* had increased fecundity compared to uninfected counterparts. Although the number of eggs decreased with age, RoWV-1 (+) *D. melanogaster* laid more eggs than their uninfected counterparts. Supplementary Fig. S2B also clarifies that RoWV-1 will increase the number *D. melanogaster* eggs when observed over 7 days. In addition, total fecundity recorded over 30 days following infection in mated females significantly increased in the RoWV-1 (+) *Drosophila* colony (Supplementary Fig. S2C). However, the fecundity of *P. vindemmiae* wasps was not significantly different regarding offspring number, mature egg number, or ovarioles. Combined with the results of virus tissue distribution, we hypothesize that this phenomenon is closely related to the high viral load in fat bodies and ovaries. In *P. vindemmiae* wasps, RoWV-1 replicated at low viral titers after the larva pupated. On the contrary, *D. melanogaster* contained large amounts of viral particles. There are several examples for fecundity increases in invertebrates subsequent to viral infection [64–67]. For our study, we hypothesize that the increase in fecundity could be related to RoWV-1 localization in follicular cells and fat bodies, which are involved in the synthesis of vitellogenin [68, 69]. DCV causes intestinal obstruction and affects the visceral muscles surrounding the crop, a bilobed extensible sac found in the abdomen of Diptera that is used as a reservoir for nutrients [70]. Therefore, the change in ovariole number is most likely attributable to a starvation response due to gut damage. Interestingly, DCV infection of *D. melanogaster* results in accelerated larval development but also causes mortality [39, 71], whereas in our study, RoWV-1 infection increased the developmental duration of *D. melanogaster* by increasing the pupal duration. Female fecundity increases with body size and size increases with development time [72]. Such an effect may be beneficial for *P. vindemmiae* wasps, as an extended pupal period of *D. melanogaster* provides a longer time period for the wasps to find their pupal hosts and the increased fecundity of the hosts possibly provides more *D. melanogaster* to the *P. vindemmiae* wasps for parasitization.

Generally, when resistance is possible and virulence is low, reducing investment in costly reproduction may provide more resources with which to mount an immune response. When resistance is futile, because either resistance is impossible or virulence is high, hosts may still compensate by reproducing early and forgoing a costly immune battle [66]. In our study, we found the eclosion rate of infected *D. melanogaster* to be lower than that of uninfected *D. melanogaster* (Fig. 8F). Therefore, the increased fecundity may represent an effective response to decrease the eclosion rate. In our study, RoWV-1 infection increases the fecundity and development duration of *D. melanogaster* but had no obvious effects on *P. vindemmiae*, possibly as a resolute of co-evolution. The parasitoid wasps provide a place in which RoWV-1 can replicate rapidly, while RoWV-1 induces *D. melanogaster* to provide more pupae for *P. vindemmiae* wasps.

Until this study was conducted, the interaction between *P. vindemmiae* wasps and *D. melanogaster* was not well understood. To address this gap, we assessed the fitness of an insect RNA virus (RoWV-1), a parasitic wasp (*P. vindemmiae*), and a host insect (*D. melanogaster*). The egg load of *D. melanogaster* was higher when infected with RoWV-1. This result suggests that RoWV-1 contributed to the increase in *D. melanogaster* fecundity in response to providing more hosts for the parasitoid wasps. On the other hand, a reduced eclosion rate is a population threat for *D. melanogaster*. Under the pressure of survival selection, more offspring can be produced to maintain a stable population. Surprisingly, we could link the decrease in eclosion rate to RoWV-1.

Discovery of increasing numbers of RNA viruses in parasitoid wasps has prompted investigation to gradually unravel the consequences of these infections. In this study, we discovered a novel RNA virus in *P. vindemmiae* wasps, RoWV-1, which is transmitted horizontally. Although present in different wasp tissues, RoWV-1 infection appears not to be detrimental to the wasps. Interestingly, RoWV-1 can also infect *D. melanogaster*, the hosts of the wasps, creating a rather complicated situation of cross-transmission of RoWV-1 among wasps and *D. melanogaster*. Infection of *D. melanogaster* resulted in prolonged pupal developmental and increased fecundity of the flies, suggesting that RoWV-1 is a crucial factor in the flies’ lifecycle. Our study provides new insight for studying the interaction among viruses, parasites, and hosts. *P. vindemmiae* wasps and the *D. melanogaster* hosts provide a good model for studying the cross-species transmission of viruses. RoWV-1 could infect wasp offspring via feeding on *D. melanogaster* pupae, suggesting that other parasitoid wasps could be infected in similar ways by similar viruses. Once better understood, these virus–host relationships could be exploited in pest control.

## Supplementary information

Supplementary figure and table legends

Supplementary Fig. S1

Supplementary Fig. S2

Supplementary Fig. S3

Supplementary Fig. S4

Supplementary Fig. S5

Supplementary Fig. S6

Supplementary Fig. S7

Supplementary Fig. S8

Supplementary Table S1

Supplementary Table S2

Supplementary Table S3

Supplementary Table S4

Supplementary Table S5
