# Supplementary material for: A novel cripavirus of an ectoparasitoid wasp increases pupal duration and fecundity of the wasp’s Drosophila melanogaster host
Source: ISME J. 2021 May 18;15(11):3239–57. doi: 10.1038/s41396-021-01005-w (PMC8528920; doi:10.1038/s41396-021-01005-w)
Supplement: Supplementary file 1 — Supplementary figure and table legends [file 41396_2021_1005_MOESM1_ESM.docx]

## Supplementary Figure and Table Legends

**Supplementary Fig. S1: Schematic diagram of fecundity device.**

The fecundity device shown here is a large sterilized plastic tube that provides activity space for the *Drosophila melanogaster*. A sponge can be used to anesthetize the flies after soaking with anesthetic and also maintains air flow. Yeast extract serves as a food source. Grape juice medium (500 mL deionized distilled water, 500 mL grape juice (Weichuan, Shanghai, China), 30 g agar (Sangon Biotech), 10 mL anhydrous ethanol absolute (Sinopharm Chemical Reagent, Shanghai, China), and 10 mL glacial acetic acid (Sinopharm Chemical Reagent)) facilitates egg collection. Grape juice medium was changed every 12 h.

**Supplementary Fig. S2. Rondani’s wasp virus 1 (RoWV-1) increases fecundity.**

**A** RoWV-1 detection with specific primers PVDA-1/PVDS-1 and PVDA‑2/PVDS-2 in *Pachycrepoideus vindemmiae* raised in a laboratory from Anhui Province. **B** Comparison of RoWV-1-infected (+) and uninfected (-) *D. melanogaster* colonies regarding fecundity every 12 h **C** and total fecundity over 7 d (n=3 independent samples). **D** Total fecundity recorded for 30 d following systemic infection in mated females (n=17 independent samples). Data represent means ± standard error of mean (SEM). Statistical significance (*t*-test) is indicated by asterisks: *, *p* < 0.05; **, *p* < 0.01; ***, *p* < 0.001.

**Supplementary Fig. S3: Rondani’s wasp virus 1 (RoWV-1) particles.**

Negatively stained transmission electron micrographs of purified RoWV-1 particles (black boxes).

**Supplementary Fig. S4: Vertical transmission of Rondani’s wasp virus 1 (RoWV-1) in *Pachycrepoideus vindemmiae* wasps.**

RoWV-1 infection as determined by RT-PCR with specific primers PVDA-1/PVDS-1 and PVDA-2/PVDS-2 followed by gel electrophoresis. **A ♀−/♂−**, **B ♀+/♂+**, **C ♀+/♂−**, and **D ♀−/♂+**. A1, B1, C1, and D1: in 10 single larvae. A2, B2, C2, and D2: in 10 single female pupae. A3, B3, C3, and D3: in 10 single male pupae. A4, B4, C4, and D4: in 10 single female adults. A5, B5, C5, and D5: in 10 single male adults.

**Supplementary Fig. S5: Vertical transmission of Rondani’s wasp virus 1 (RoWV-1) in** ***Drosophila melanogaster*.**

RoWV-1 infection as determined by RT-PCR with specific primers PVDA-1/PVDS-1 and PVDA-2/PVDS-2 followed by gel electrophoresis. **A** **♀−/♂−,** **B ♀+/♂+, C ♀+/♂−** and **D ♀−/♂+**. A1, B1, C1, and D1: in 10 single larvae. A2, B2, C2, and D2: in 10 single pupae. A3, B3, C3, and D3: in 10 single female adults. A4, B4, C4, and D4: in 10 single male adults.

Supplementary Fig. S6: Rondani’s wasp virus 1 (RoWV-1) is transmitted horizontally, but not vertically, by *Drosophila melanogaster*.

**A** Vertical RoWV-1 transmission to the offspring (F_1_) and **B** horizontal RoWV-1 transmission from RoWV-1 (-) and RoWV-1 (+) parent *D. melanogaster* (F_0_). RoWV-1 infection was determined by RT-PCR with specific primers PVDA-1/PVDS-1 and PVDA‑2/PVDS‑2 followed by gel electrophoresis at the indicated days. **C** Analysis of horizontal RoWV-1 transmission among *D. melanogaster* in seven samples per developmental stage. RoWV-1 was detected as in B.

Supplementary Fig. S7. Rondani’s wasp virus 1 (RoWV-1) transmits bidirectionally between parasitoid wasp and *Drosophila melanogaster.*

**A** Horizontal RoWV-1 transmission from RoWV-1 (+) wasps to RoWV-1 (-) *D. melanogaster* pupae. Lines 1–8: RT-PCR detection of RoWV-1 in the individual fly pupae that were parasitized by the RoWV-1 (+) wasps for 24 h. Line 9: the fly pupae that were not parasitized. Line 10: the ddH_2_0 control i.e., without cDNA from the fly pupae. **B** Horizontal RoWV-1 transmission from the RoWV-1 (+) flies to RoWV-1 (-) wasp larvae. The RoWV-1 (-) wasp young larvae were individually placed into the RoWV-1 (+) flies’ pupae until reaching mature stage and then collected for RNA extraction and RT-PCR detection. Lines 1–10: RT-PCR detection of RoWV-1 in the individual mature wasp larva. Line 11: the mature fly pupa. Line 12: the ddH_2_0 control i.e. without cDNA from the mature fly pupae. **C** Horizontal RoWV-1 transmission from the RoWV-1 (+) flies to RoWV-1 (-) wasp. RoWV-1 infection was determined by RT-PCR with specific primers followed by gel electrophoresis.

**Supplementary Fig. S8: Phylogenetic analysis of Rondani’s wasp virus 1 (RoWV-1).**

Phylogenetic trees comparing RoWV-1 to other cripaviruses and aparaviruses. **A** RdRp amino acid residues. **B** Capsid (ORF2) protein amino acid residues. **C** Complete genome. RoWV-1 is indicated by a red star. Bootstrap values represent 1,000 replicates. Virus names and protein accession numbers are listed Supplementary Table S2.

**Supplementary Table S1: Primers for viral genome sequencing.**

**Supplementary Table S2: Virus names, abbreviations, and viral protein sequence accession numbers.**

**Supplementary Table S3:** **Information about the transcriptome.**

**Supplementary Table S4: dsRBD homologs.**

**Supplementary Table S5: RoWV-1 detection in wild *D. melanogaster* originating from various Chinese provinces.**
