# Supplementary figures and images for: A novel cripavirus of an ectoparasitoid wasp increases pupal duration and fecundity of the wasp’s Drosophila melanogaster host
Source: ISME J. 2021 May 18;15(11):3239–57. doi: 10.1038/s41396-021-01005-w (PMC8528920; doi:10.1038/s41396-021-01005-w)

Supplementary Fig. S1

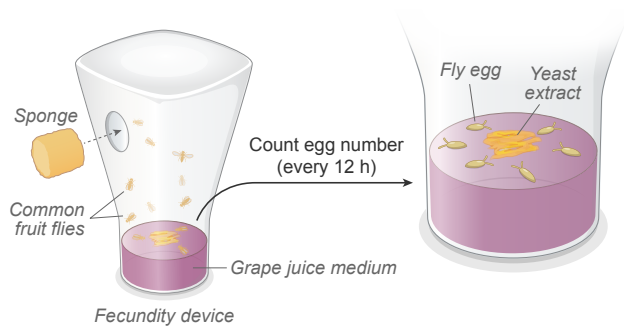

Supplement: Supplementary file 2 — Supplementary Fig. S1 [file 41396_2021_1005_MOESM2_ESM.pdf]

Supplementary Fig. S2

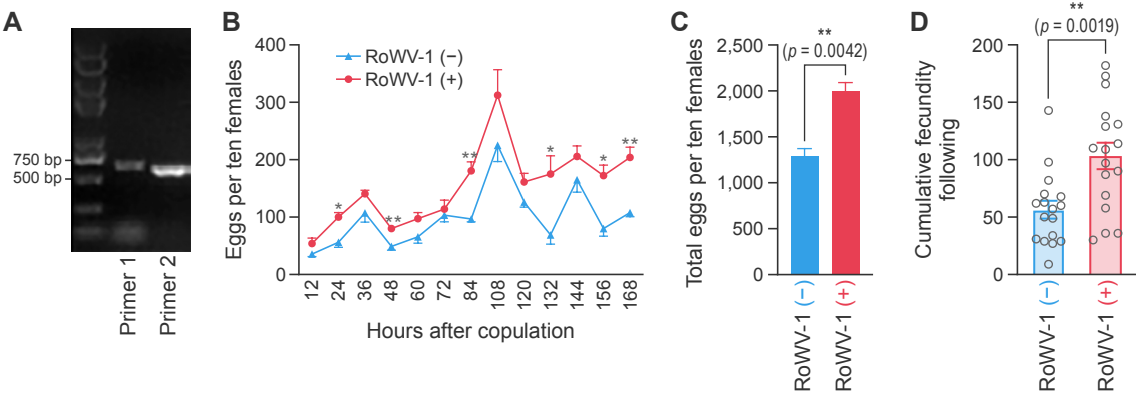

Supplement: Supplementary file 3 — Supplementary Fig. S2 [file 41396_2021_1005_MOESM3_ESM.pdf]

Supplementary Fig. S3

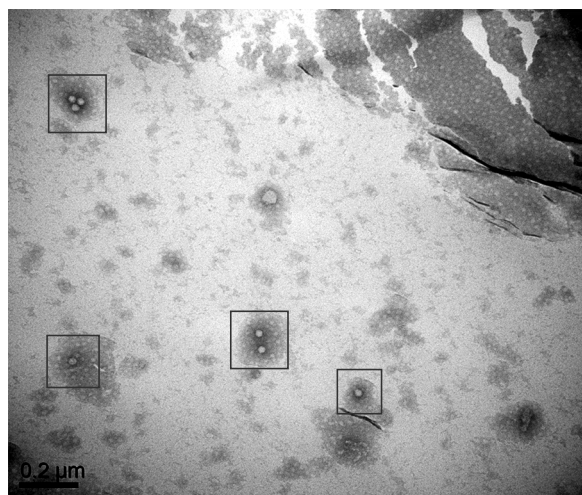

Supplement: Supplementary file 4 — Supplementary Fig. S3 [file 41396_2021_1005_MOESM4_ESM.pdf]

Supplementary Fig. S4

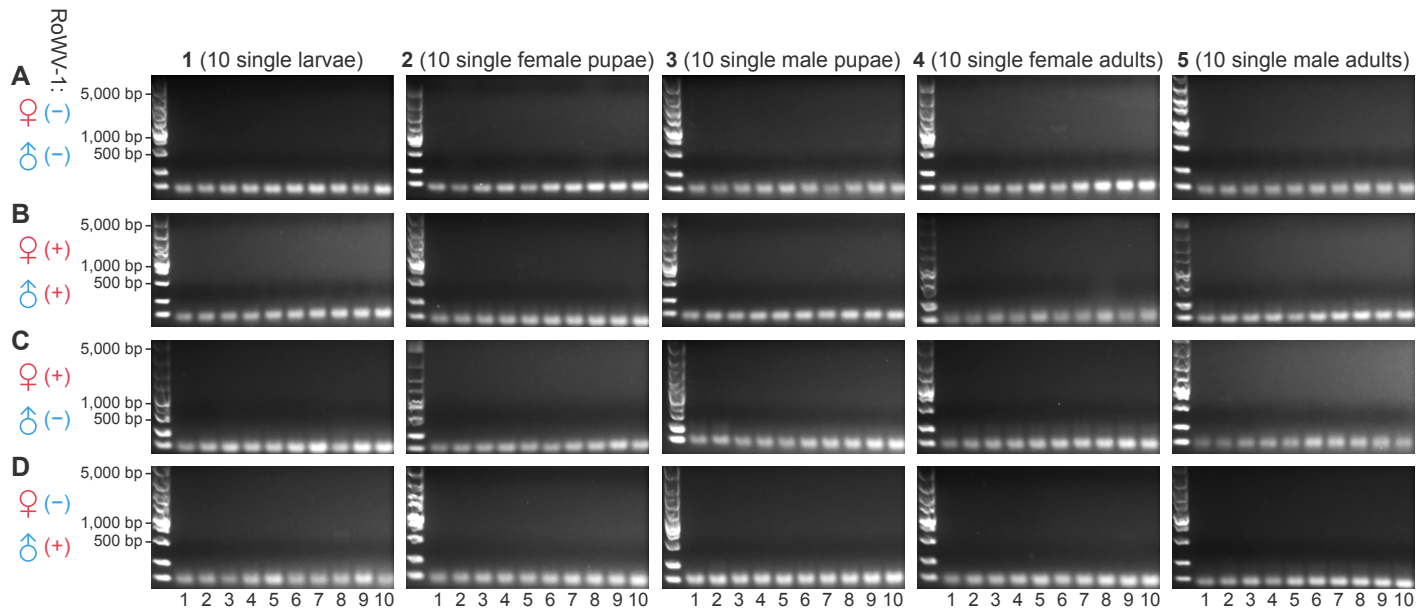

Supplement: Supplementary file 5 — Supplementary Fig. S4 [file 41396_2021_1005_MOESM5_ESM.pdf]

Fig. S5

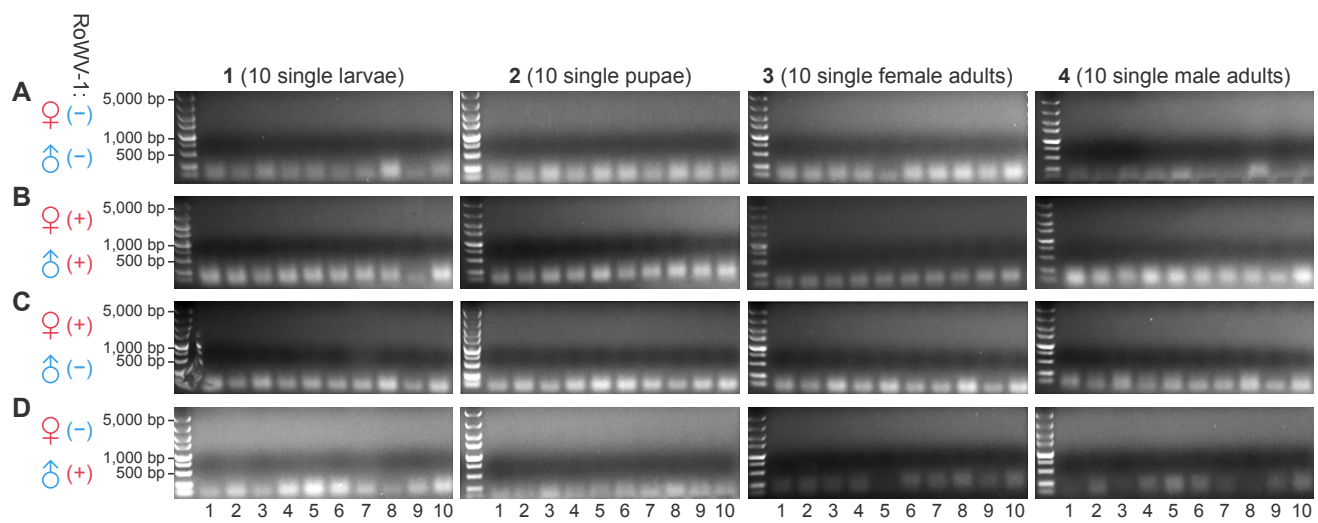

Supplement: Supplementary file 6 — Supplementary Fig. S5 [file 41396_2021_1005_MOESM6_ESM.pdf]

Supplementary Fig. S6

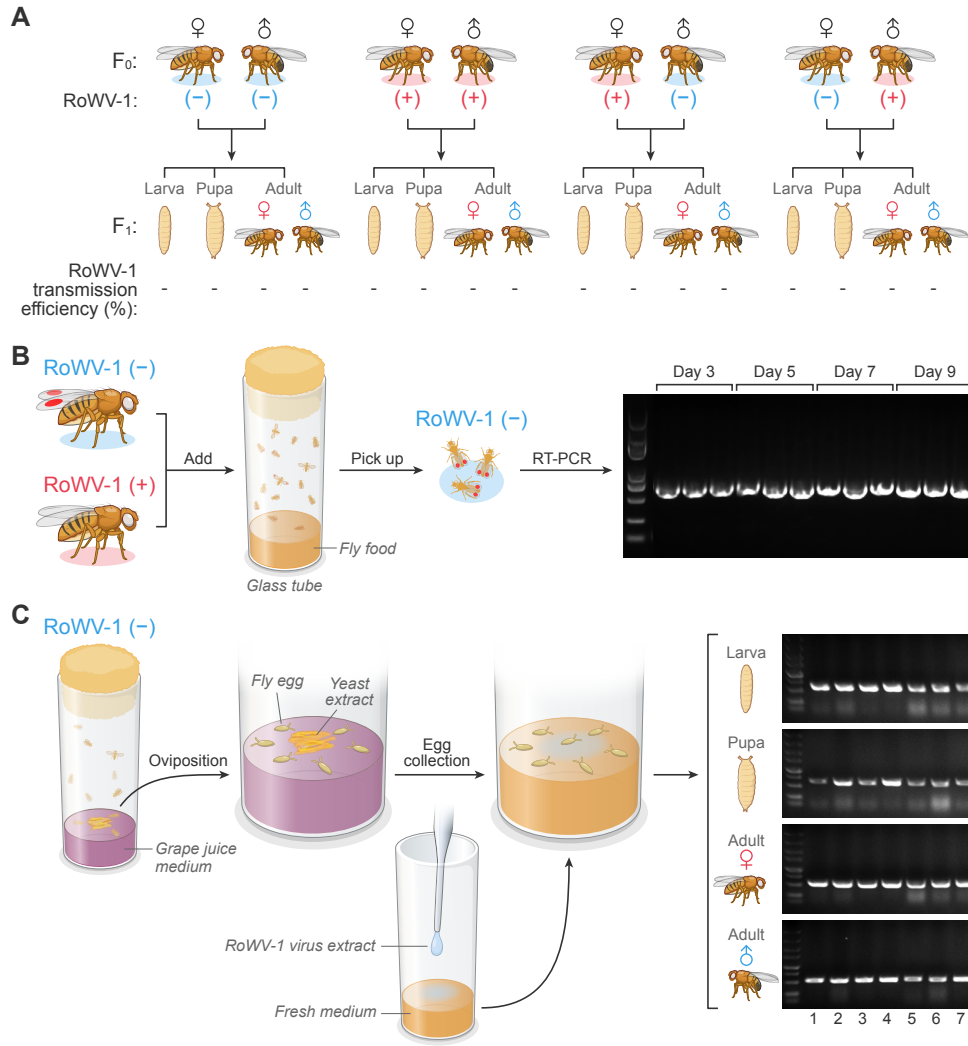

Supplement: Supplementary file 7 — Supplementary Fig. S6 [file 41396_2021_1005_MOESM7_ESM.pdf]

Supplementary Fig. S7

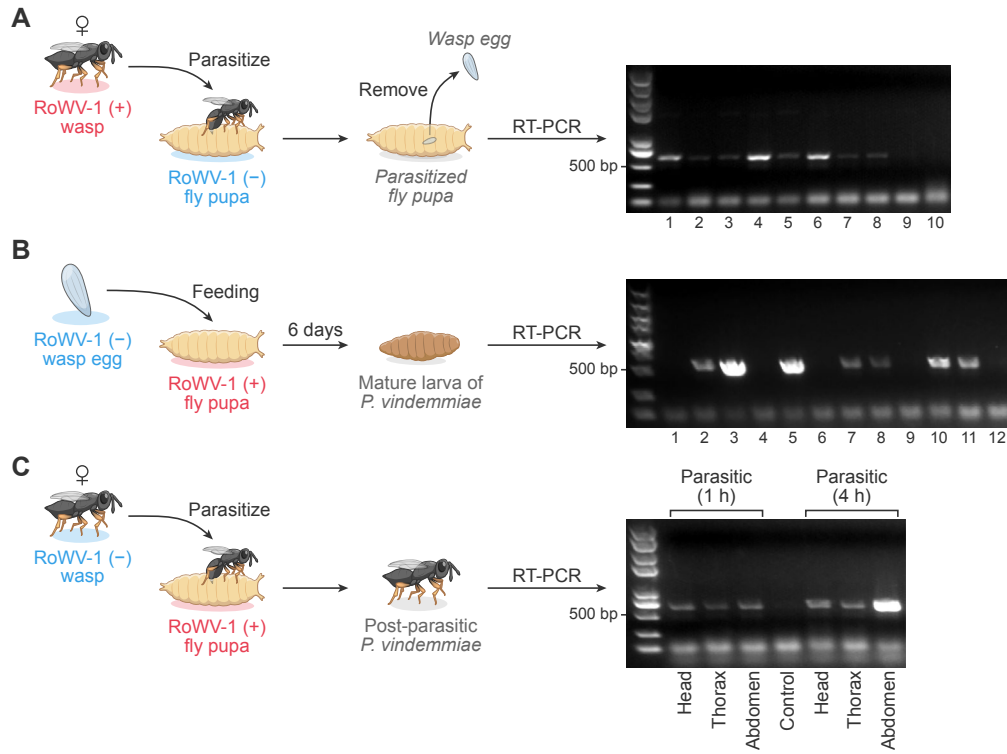

Supplement: Supplementary file 8 — Supplementary Fig. S7 [file 41396_2021_1005_MOESM8_ESM.pdf]

Supplementary Fig. S8

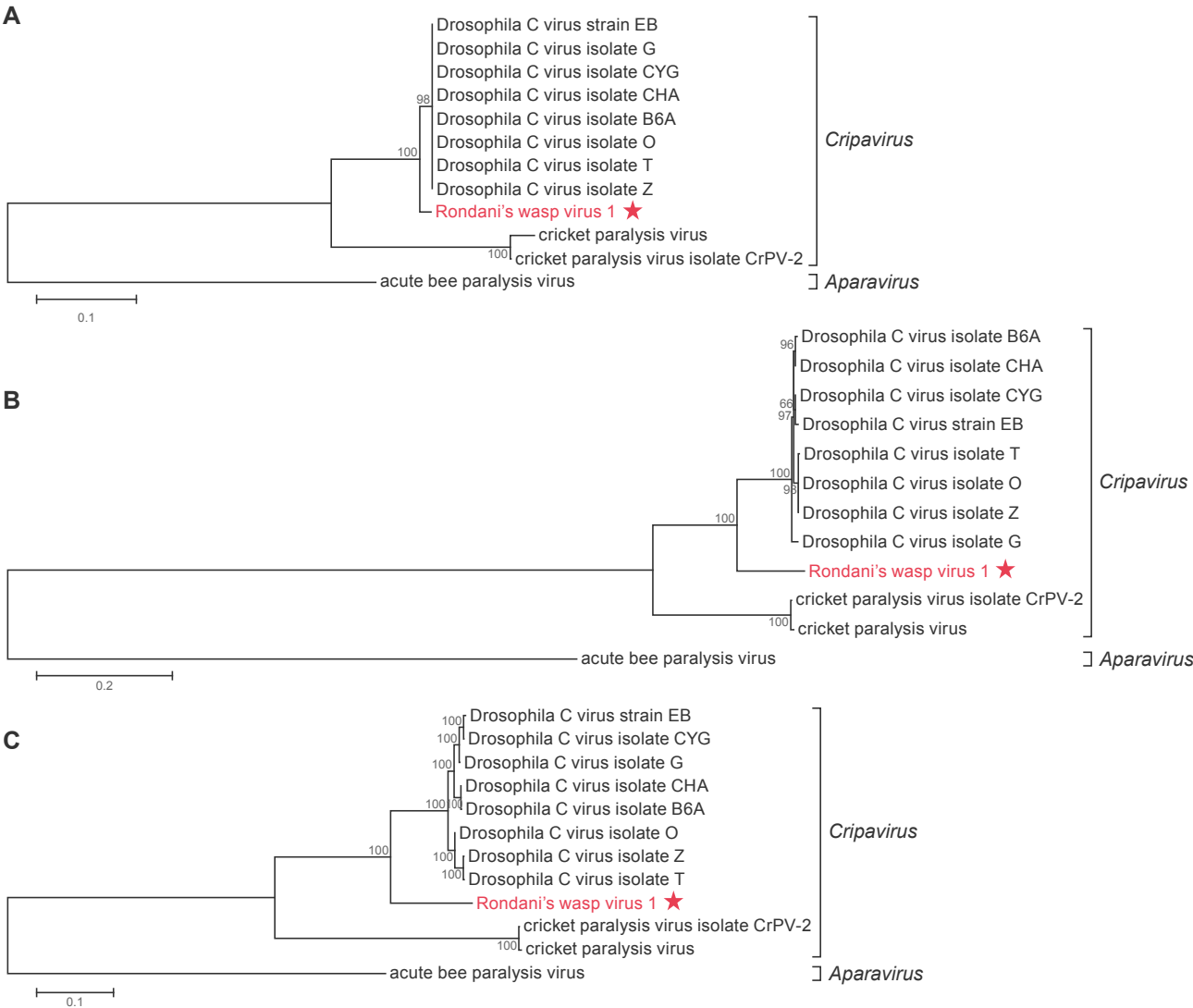

Supplement: Supplementary file 9 — Supplementary Fig. S8 [file 41396_2021_1005_MOESM9_ESM.pdf]
